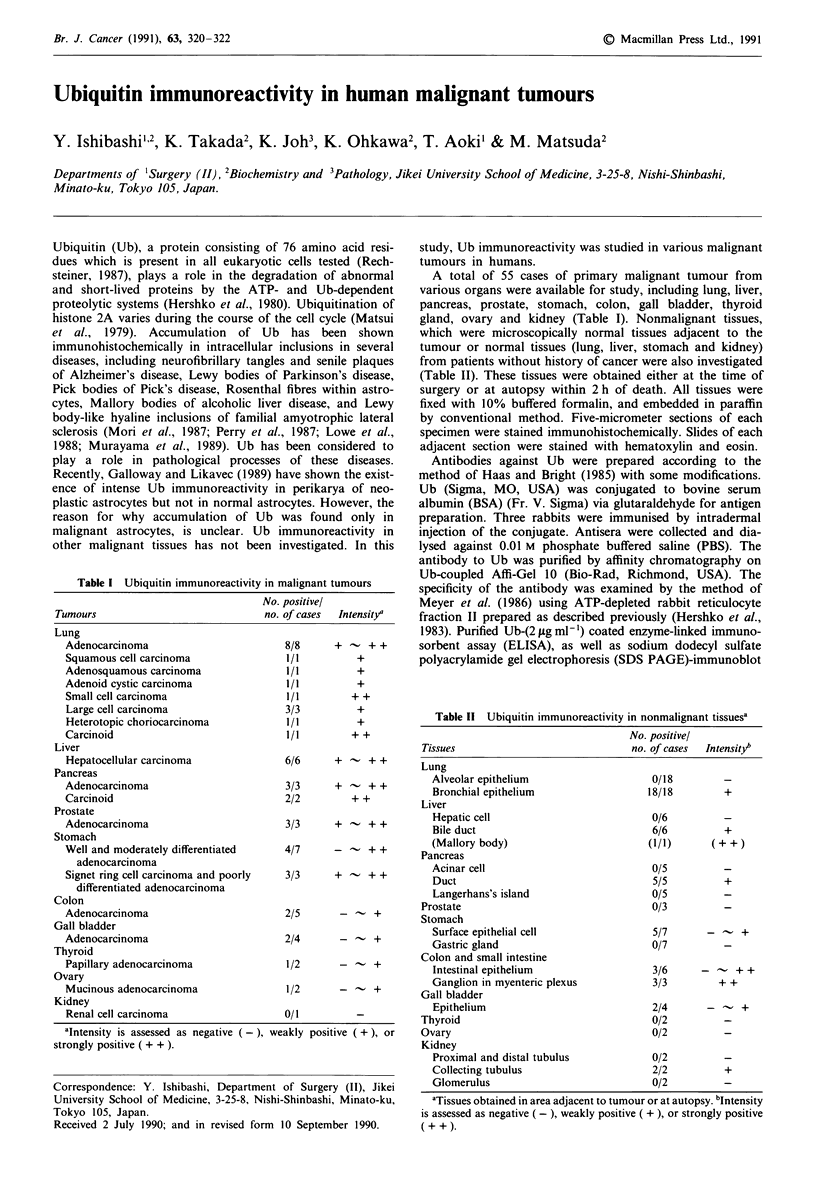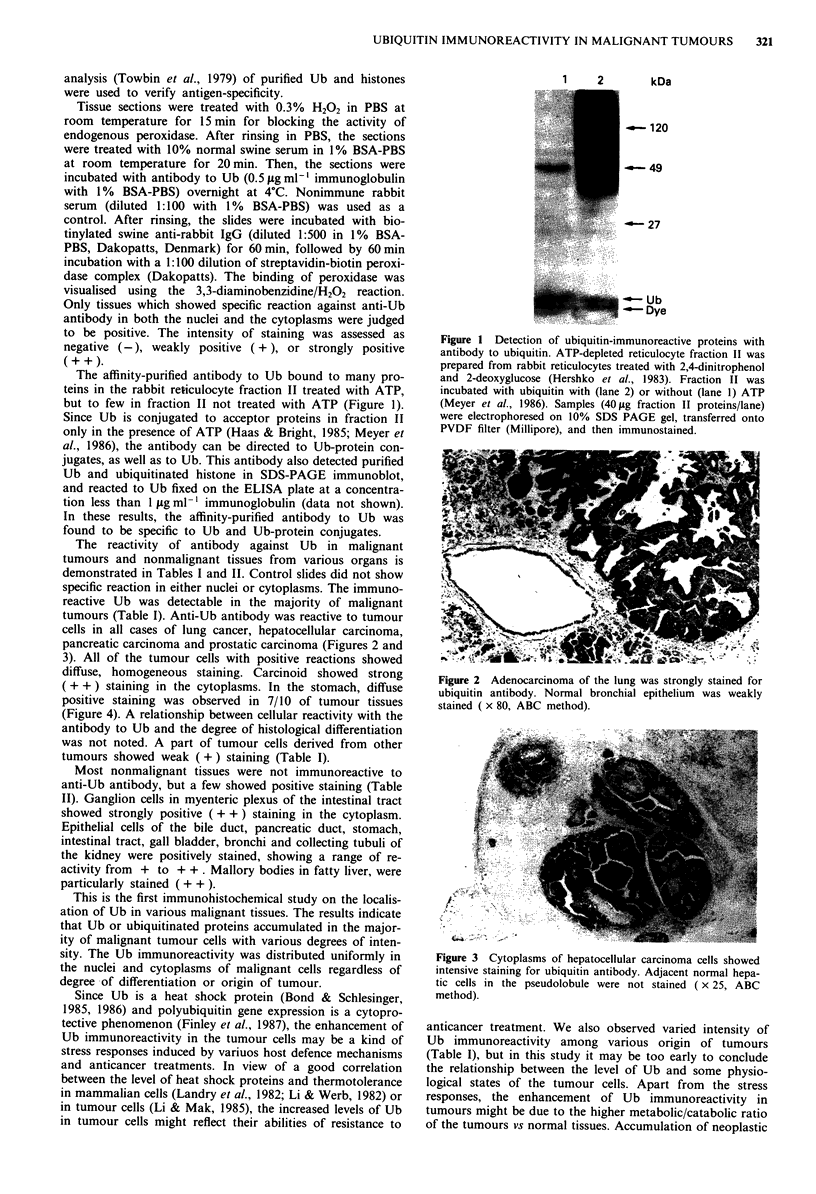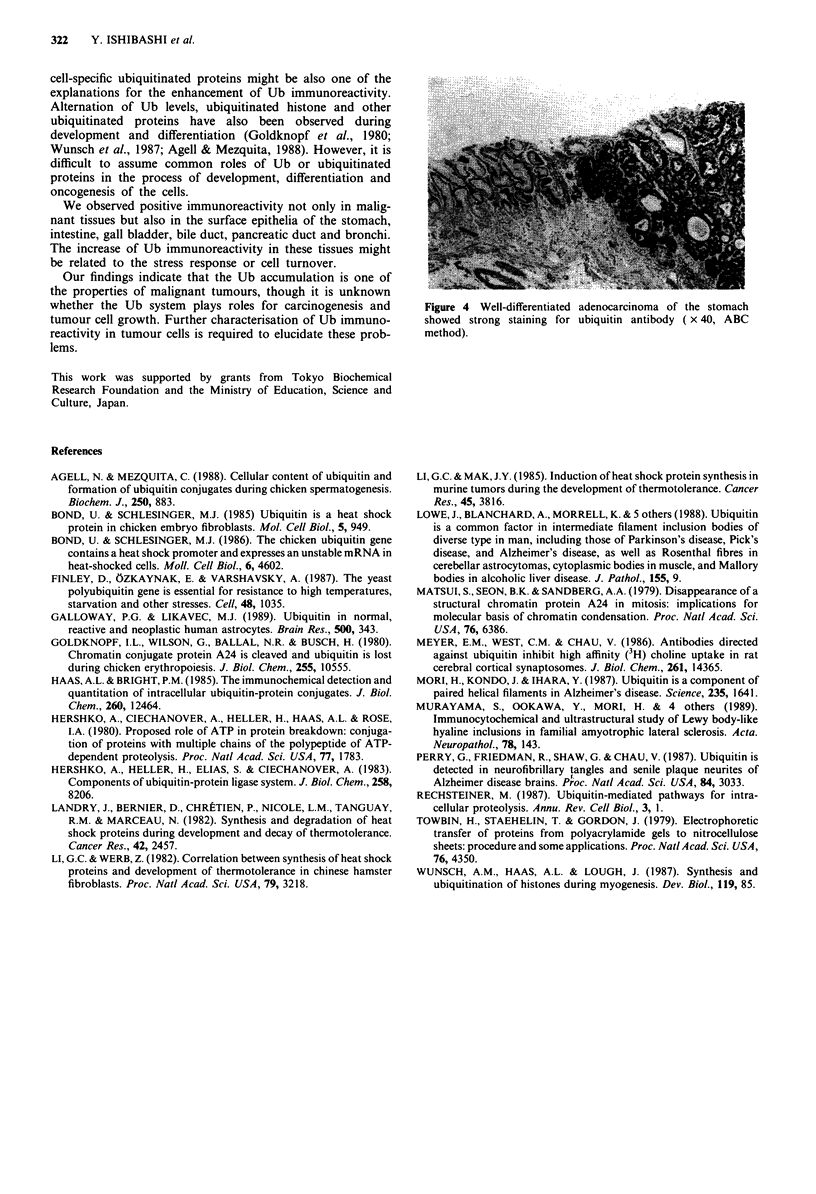# Ubiquitin immunoreactivity in human malignant tumours.

**DOI:** 10.1038/bjc.1991.75

**Published:** 1991-02

**Authors:** Y. Ishibashi, K. Takada, K. Joh, K. Ohkawa, T. Aoki, M. Matsuda

**Affiliations:** Department of Surgery (II), Jikei University School of Medicine, Tokyo, Japan.

## Abstract

**Images:**


 © Macmillan Press Ltd., 1991

### Ubiquitin immunoreactivity in human malignant tumours

Y. Ishibashi[1,2], K. Takada[2], K. Joh[3], K. Ohkawa[2], T. Aoki[1] & M. Matsuda[2]

*Departments of [1]Surgery (II), [2]Biochemistry and [3]Pathology, Jikei University School of Medicine, 3-25-8, Nishi-Shinbashi, Minato-ku, Tokyo 105, Japan.*

Ubiquitin (Ub), a protein consisting of 76 amino acid residues which is present in all eukaryotic cells tested (Rechsteiner, 1987), plays a role in the degradation of abnormal and short-lived proteins by the ATP- and Ub-dependent proteolytic systems (Hershko *et al.*, 1980). Ubiquitination of histone 2A varies during the course of the cell cycle (Matsui *et al.*, 1979). Accumulation of Ub has been shown immunohistochemically in intracellular inclusions in several diseases, including neurofibrillary tangles and senile plaques of Alzheimer's disease, Lewy bodies of Parkinson's disease, Pick bodies of Pick's disease, Rosenthal fibres within astrocytes, Mallory bodies of alcoholic liver disease, and Lewy body-like hyaline inclusions of familial amyotrophic lateral sclerosis (Mori *et al.*, 1987; Perry *et al.*, 1987; Lowe *et al.*, 1988; Murayama *et al.*, 1989). Ub has been considered to play a role in pathological processes of these diseases. Recently, Galloway and Likavec (1989) have shown the existence of intense Ub immunoreactivity in perikarya of neoplastic astrocytes but not in normal astrocytes. However, the reason for why accumulation of Ub was found only in malignant astrocytes, is unclear. Ub immunoreactivity in other malignant tissues has not been investigated. In this study, Ub immunoreactivity was studied in various malignant tumours in humans.

A total of 55 cases of primary malignant tumour from various organs were available for study, including lung, liver, pancreas, prostate, stomach, colon, gall bladder, thyroid gland, ovary and kidney (Table I). Nonmalignant tissues, which were microscopically normal tissues adjacent to the tumour or normal tissues (lung, liver, stomach and kidney) from patients without history of cancer were also investigated (Table II). These tissues were obtained either at the time of surgery or at autopsy within 2 h of death. All tissues were fixed with 10% buffered formalin, and embedded in paraffin by conventional method. Five-micrometer sections of each specimen were stained immunohistochemically. Slides of each adjacent section were stained with hematoxylin and eosin.

Antibodies against Ub were prepared according to the method of Haas and Bright (1985) with some modifications. Ub (Sigma, MO, USA) was conjugated to bovine serum albumin (BSA) (Fr. V. Sigma) via glutaraldehyde for antigen preparation. Three rabbits were immunised by intradermal injection of the conjugate. Antisera were collected and dialysed against 0.01 M phosphate buffered saline (PBS). The antibody to Ub was purified by affinity chromatography on Ub-coupled Affi-Gel 10 (Bio-Rad, Richmond, USA). The specificity of the antibody was examined by the method of Meyer *et al.* (1986) using ATP-depleted rabbit reticulocyte fraction II prepared as described previously (Hershko *et al.*, 1983). Purified Ub-(2 $\mu g\ ml^{-1}$) coated enzyme-linked immunosorbent assay (ELISA), as well as sodium dodecyl sulfate polyacrylamide gel electrophoresis (SDS PAGE)-immunoblot

**Table I** Ubiquitin immunoreactivity in malignant tumours

| *Tumours* | *No. positive/ no. of cases* | *Intensity*[a] |
|---|---|---|
| Lung | | |
| Adenocarcinoma | 8/8 | + ~ ++ |
| Squamous cell carcinoma | 1/1 | + |
| Adenosquamous carcinoma | 1/1 | + |
| Adenoid cystic carcinoma | 1/1 | + |
| Small cell carcinoma | 1/1 | ++ |
| Large cell carcinoma | 3/3 | + |
| Heterotopic choriocarcinoma | 1/1 | + |
| Carcinoid | 1/1 | ++ |
| Liver | | |
| Hepatocellular carcinoma | 6/6 | + ~ ++ |
| Pancreas | | |
| Adenocarcinoma | 3/3 | + ~ ++ |
| Carcinoid | 2/2 | ++ |
| Prostate | | |
| Adenocarcinoma | 3/3 | + ~ ++ |
| Stomach | | |
| Well and moderately differentiated adenocarcinoma | 4/7 | – ~ ++ |
| Signet ring cell carcinoma and poorly differentiated adenocarcinoma | 3/3 | + ~ ++ |
| Colon | | |
| Adenocarcinoma | 2/5 | – ~ + |
| Gall bladder | | |
| Adenocarcinoma | 2/4 | – ~ + |
| Thyroid | | |
| Papillary adenocarcinoma | 1/2 | – ~ + |
| Ovary | | |
| Mucinous adenocarcinoma | 1/2 | – ~ + |
| Kidney | | |
| Renal cell carcinoma | 0/1 | – |

[a]Intensity is assessed as negative (–), weakly positive (+), or strongly positive (++).

**Table II** Ubiquitin immunoreactivity in nonmalignant tissues[a]

| *Tissues* | *No. positive/ no. of cases* | *Intensity*[b] |
|---|---|---|
| Lung | | |
| Alveolar epithelium | 0/18 | – |
| Bronchial epithelium | 18/18 | + |
| Liver | | |
| Hepatic cell | 0/6 | – |
| Bile duct | 6/6 | + |
| (Mallory body) | (1/1) | (++) |
| Pancreas | | |
| Acinar cell | 0/5 | – |
| Duct | 5/5 | + |
| Langerhans's island | 0/5 | – |
| Prostate | 0/3 | – |
| Stomach | | |
| Surface epithelial cell | 5/7 | – ~ + |
| Gastric gland | 0/7 | – |
| Colon and small intestine | | |
| Intestinal epithelium | 3/6 | – ~ ++ |
| Ganglion in myenteric plexus | 3/3 | ++ |
| Gall bladder | | |
| Epithelium | 2/4 | – ~ + |
| Thyroid | 0/2 | – |
| Ovary | 0/2 | – |
| Kidney | | |
| Proximal and distal tubulus | 0/2 | – |
| Collecting tubulus | 2/2 | + |
| Glomerulus | 0/2 | – |

[a]Tissues obtained in area adjacent to tumour or at autopsy. [b]Intensity is assessed as negative (–), weakly positive (+), or strongly positive (++).

Correspondence: Y. Ishibashi, Department of Surgery (II), Jikei University School of Medicine, 3-25-8, Nishi-Shinbashi, Minato-ku, Tokyo 105, Japan.
Received 2 July 1990; and in revised form 10 September 1990.

analysis (Towbin *et al.*, 1979) of purified Ub and histones were used to verify antigen-specificity.

Tissue sections were treated with 0.3% $H_2O_2$ in PBS at room temperature for 15 min for blocking the activity of endogenous peroxidase. After rinsing in PBS, the sections were treated with 10% normal swine serum in 1% BSA-PBS at room temperature for 20 min. Then, the sections were incubated with antibody to Ub (0.5 $\mu g\,ml^{-1}$ immunoglobulin with 1% BSA-PBS) overnight at 4°C. Nonimmune rabbit serum (diluted 1:100 with 1% BSA-PBS) was used as a control. After rinsing, the slides were incubated with biotinylated swine anti-rabbit IgG (diluted 1:500 in 1% BSA-PBS, Dakopatts, Denmark) for 60 min, followed by 60 min incubation with a 1:100 dilution of streptavidin-biotin peroxidase complex (Dakopatts). The binding of peroxidase was visualised using the 3,3-diaminobenzidine/$H_2O_2$ reaction. Only tissues which showed specific reaction against anti-Ub antibody in both the nuclei and the cytoplasms were judged to be positive. The intensity of staining was assessed as negative (−), weakly positive (+), or strongly positive (++).

The affinity-purified antibody to Ub bound to many proteins in the rabbit reticulocyte fraction II treated with ATP, but to few in fraction II not treated with ATP (Figure 1). Since Ub is conjugated to acceptor proteins in fraction II only in the presence of ATP (Haas & Bright, 1985; Meyer *et al.*, 1986), the antibody can be directed to Ub-protein conjugates, as well as to Ub. This antibody also detected purified Ub and ubiquitinated histone in SDS-PAGE immunoblot, and reacted to Ub fixed on the ELISA plate at a concentration less than 1 $\mu g\,ml^{-1}$ immunoglobulin (data not shown). In these results, the affinity-purified antibody to Ub was found to be specific to Ub and Ub-protein conjugates.

The reactivity of antibody against Ub in malignant tumours and nonmalignant tissues from various organs is demonstrated in Tables I and II. Control slides did not show specific reaction in either nuclei or cytoplasms. The immunoreactive Ub was detectable in the majority of malignant tumours (Table I). Anti-Ub antibody was reactive to tumour cells in all cases of lung cancer, hepatocellular carcinoma, pancreatic carcinoma and prostatic carcinoma (Figures 2 and 3). All of the tumour cells with positive reactions showed diffuse, homogeneous staining. Carcinoid showed strong (++) staining in the cytoplasms. In the stomach, diffuse positive staining was observed in 7/10 of tumour tissues (Figure 4). A relationship between cellular reactivity with the antibody to Ub and the degree of histological differentiation was not noted. A part of tumour cells derived from other tumours showed weak (+) staining (Table I).

Most nonmalignant tissues were not immunoreactive to anti-Ub antibody, but a few showed positive staining (Table II). Ganglion cells in myenteric plexus of the intestinal tract showed strongly positive (++) staining in the cytoplasm. Epithelial cells of the bile duct, pancreatic duct, stomach, intestinal tract, gall bladder, bronchi and collecting tubuli of the kidney were positively stained, showing a range of reactivity from + to ++. Mallory bodies in fatty liver, were particularly stained (++).

This is the first immunohistochemical study on the localisation of Ub in various malignant tissues. The results indicate that Ub or ubiquitinated proteins accumulated in the majority of malignant tumour cells with various degrees of intensity. The Ub immunoreactivity was distributed uniformly in the nuclei and cytoplasms of malignant cells regardless of degree of differentiation or origin of tumour.

Since Ub is a heat shock protein (Bond & Schlesinger, 1985, 1986) and polyubiquitin gene expression is a cytoprotective phenomenon (Finley *et al.*, 1987), the enhancement of Ub immunoreactivity in the tumour cells may be a kind of stress responses induced by variuos host defence mechanisms and anticancer treatments. In view of a good correlation between the level of heat shock proteins and thermotolerance in mammalian cells (Landry *et al.*, 1982; Li & Werb, 1982) or in tumour cells (Li & Mak, 1985), the increased levels of Ub in tumour cells might reflect their abilities of resistance to

**Figure 1** Detection of ubiquitin-immunoreactive proteins with antibody to ubiquitin. ATP-depleted reticulocyte fraction II was prepared from rabbit reticulocytes treated with 2,4-dinitrophenol and 2-deoxyglucose (Hershko *et al.*, 1983). Fraction II was incubated with ubiquitin with (lane 2) or without (lane 1) ATP (Meyer *et al.*, 1986). Samples (40 μg fraction II proteins/lane) were electrophoresed on 10% SDS PAGE gel, transferred onto PVDF filter (Millipore), and then immunostained.

**Figure 2** Adenocarcinoma of the lung was strongly stained for ubiquitin antibody. Normal bronchial epithelium was weakly stained (×80, ABC method).

**Figure 3** Cytoplasms of hepatocellular carcinoma cells showed intensive staining for ubiquitin antibody. Adjacent normal hepatic cells in the pseudolobule were not stained (×25, ABC method).

anticancer treatment. We also observed varied intensity of Ub immunoreactivity among various origin of tumours (Table I), but in this study it may be too early to conclude the relationship between the level of Ub and some physiological states of the tumour cells. Apart from the stress responses, the enhancement of Ub immunoreactivity in tumours might be due to the higher metabolic/catabolic ratio of the tumours *vs* normal tissues. Accumulation of neoplastic cell-specific ubiquitinated proteins might be also one of the explanations for the enhancement of Ub immunoreactivity. Alternation of Ub levels, ubiquitinated histone and other ubiquitinated proteins have also been observed during development and differentiation (Goldknopf et al., 1980; Wunsch et al., 1987; Agell & Mezquita, 1988). However, it is difficult to assume common roles of Ub or ubiquitinated proteins in the process of development, differentiation and oncogenesis of the cells.

We observed positive immunoreactivity not only in malignant tissues but also in the surface epithelia of the stomach, intestine, gall bladder, bile duct, pancreatic duct and bronchi. The increase of Ub immunoreactivity in these tissues might be related to the stress response or cell turnover.

Our findings indicate that the Ub accumulation is one of the properties of malignant tumours, though it is unknown whether the Ub system plays roles for carcinogenesis and tumour cell growth. Further characterisation of Ub immunoreactivity in tumour cells is required to elucidate these problems.

**Figure 4** Well-differentiated adenocarcinoma of the stomach showed strong staining for ubiquitin antibody ( × 40, ABC method).

This work was supported by grants from Tokyo Biochemical Research Foundation and the Ministry of Education, Science and Culture, Japan.

### References

AGELL, N. & MEZQUITA, C. (1988). Cellular content of ubiquitin and formation of ubiquitin conjugates during chicken spermatogenesis. *Biochem. J.*, **250**, 883.

BOND, U. & SCHLESINGER, M.J. (1985) Ubiquitin is a heat shock protein in chicken embryo fibroblasts. *Mol. Cell Biol.*, **5**, 949.

BOND, U. & SCHLESINGER, M.J. (1986). The chicken ubiquitin gene contains a heat shock promoter and expresses an unstable mRNA in heat-shocked cells. *Moll. Cell Biol.*, **6**, 4602.

FINLEY, D., ÖZKAYNAK, E. & VARSHAVSKY, A. (1987). The yeast polyubiquitin gene is essential for resistance to high temperatures, starvation and other stresses. *Cell*, **48**, 1035.

GALLOWAY, P.G. & LIKAVEC, M.J. (1989). Ubiquitin in normal, reactive and neoplastic human astrocytes. *Brain Res.*, **500**, 343.

GOLDKNOPF, I.L., WILSON, G., BALLAL, N.R. & BUSCH, H. (1980). Chromatin conjugate protein A24 is cleaved and ubiquitin is lost during chicken erythropoiesis. *J. Biol. Chem.*, **255**, 10555.

HAAS, A.L. & BRIGHT, P.M. (1985). The immunochemical detection and quantitation of intracellular ubiquitin-protein conjugates. *J. Biol. Chem.*, **260**, 12464.

HERSHKO, A., CIECHANOVER, A., HELLER, H., HAAS, A.L. & ROSE, I.A. (1980). Proposed role of ATP in protein breakdown: conjugation of proteins with multiple chains of the polypeptide of ATP-dependent proteolysis. *Proc. Natl Acad. Sci. USA*, **77**, 1783.

HERSHKO, A., HELLER, H., ELIAS, S. & CIECHANOVER, A. (1983). Components of ubiquitin-protein ligase system. *J. Biol. Chem.*, **258**, 8206.

LANDRY, J., BERNIER, D., CHRÉTIEN, P., NICOLE, L.M., TANGUAY, R.M. & MARCEAU, N. (1982). Synthesis and degradation of heat shock proteins during development and decay of thermotolerance. *Cancer Res.*, **42**, 2457.

LI, G.C. & WERB, Z. (1982). Correlation between synthesis of heat shock proteins and development of thermotolerance in chinese hamster fibroblasts. *Proc. Natl Acad. Sci. USA*, **79**, 3218.

LI, G.C. & MAK, J.Y. (1985). Induction of heat shock protein synthesis in murine tumors during the development of thermotolerance. *Cancer Res.*, **45**, 3816.

LOWE, J., BLANCHARD, A., MORRELL, K. & 5 others (1988). Ubiquitin is a common factor in intermediate filament inclusion bodies of diverse type in man, including those of Parkinson's disease, Pick's disease, and Alzheimer's disease, as well as Rosenthal fibres in cerebellar astrocytomas, cytoplasmic bodies in muscle, and Mallory bodies in alcoholic liver disease. *J. Pathol.*, **155**, 9.

MATSUI, S., SEON, B.K. & SANDBERG, A.A. (1979). Disappearance of a structural chromatin protein A24 in mitosis: implications for molecular basis of chromatin condensation. *Proc. Natl Acad. Sci. USA*, **76**, 6386.

MEYER, E.M., WEST, C.M. & CHAU, V. (1986). Antibodies directed against ubiquitin inhibit high affinity ($^3$H) choline uptake in rat cerebral cortical synaptosomes. *J. Biol. Chem.*, **261**, 14365.

MORI, H., KONDO, J. & IHARA, Y. (1987). Ubiquitin is a component of paired helical filaments in Alzheimer's disease. *Science*, **235**, 1641.

MURAYAMA, S., OOKAWA, Y., MORI, H. & 4 others (1989). Immunocytochemical and ultrastructural study of Lewy body-like hyaline inclusions in familial amyotrophic lateral sclerosis. *Acta. Neuropathol.*, **78**, 143.

PERRY, G., FRIEDMAN, R., SHAW, G. & CHAU, V. (1987). Ubiquitin is detected in neurofibrillary tangles and senile plaque neurites of Alzheimer disease brains. *Proc. Natl Acad. Sci. USA*, **84**, 3033.

RECHSTEINER, M. (1987). Ubiquitin-mediated pathways for intracellular proteolysis. *Annu. Rev. Cell Biol.*, **3**, 1.

TOWBIN, H., STAEHELIN, T. & GORDON, J. (1979). Electrophoretic transfer of proteins from polyacrylamide gels to nitrocellulose sheets: procedure and some applications. *Proc. Natl Acad. Sci. USA*, **76**, 4350.

WUNSCH, A.M., HAAS, A.L. & LOUGH, J. (1987). Synthesis and ubiquitination of histones during myogenesis. *Dev. Biol.*, **119**, 85.